# Risk Stratification Score to Predict Readmission of Patients With Acute Decompensated Cirrhosis Within 90 Days

**DOI:** 10.3389/fmed.2021.646875

**Published:** 2021-05-31

**Authors:** Xiaomei Xu, Juntao Tan, Haolin Wang, Wenlong Zhao, Bo Qin

**Affiliations:** ^1^Department of Infectious Diseases, The First Affiliated Hospital of Chongqing Medical University, Chongqing, China; ^2^Department of Gastroenterology, The Fifth People's Hospital of Chengdu, Chengdu, China; ^3^College of Medical Informatics, Chongqing Medical University, Chongqing, China; ^4^Medical Data Science Academy, Chongqing Medical University, Chongqing, China

**Keywords:** acute decompensated cirrhosis, readmission, independent predictors, nomogram, risk stratification

## Abstract

**Background and Aims:** Patients with acute decompensated (AD) cirrhosis are frequently readmitted to the hospital. An accurate predictive model for identifying high-risk patients may facilitate the development of effective interventions to reduce readmission rates.

**Methods:** This cohort study of patients with AD cirrhosis was conducted at six tertiary hospitals in China between September 2012 and December 2016 (with 705 patients in the derivation cohort) and between January 2017 and April 2020 (with 251 patients in the temporal validation cohort). Least absolute shrinkage and selection operator Cox regression was used to identify the prognostic factors and construct a nomogram. The discriminative ability, calibration, and clinical net benefit were evaluated based on the C-index, area under the curve, calibration curve, and decision curve analysis. Kaplan–Meier curves were constructed for stratified risk groups, and log-rank tests were used to determine significant differences between the curves.

**Results:** Among 956 patients, readmission rates were 24.58, 42.99, and 51.78%, at 30, 60, and 90 days, respectively. Bacterial infection was the main reason for index hospitalization and readmission. Independent factors in the nomogram included gastrointestinal bleeding [hazard rate (HR): 2.787; 95% confidence interval (CI): 2.221–3.499], serum sodium (HR: 0.955; 95% CI: 0.933–0.978), total bilirubin (HR: 1.004; 95% CI: 1.003–1.005), and international normalized ratio (HR: 1.398; 95% CI: 1.126–1.734). For the convenience of clinicians, we provided a web-based calculator tool (https://cqykdx1111.shinyapps.io/dynnomapp/). The nomogram exhibited good discrimination ability, both in the derivation and validation cohorts. The predicted and observed readmission probabilities were calibrated with reliable agreement. The nomogram demonstrated superior net benefits over other score models. The high-risk group (nomogram score >56.8) was significantly likely to have higher rates of readmission than the low-risk group (nomogram score ≤ 56.8; *p* < 0.0001).

**Conclusions:** The nomogram is useful for assessing the probability of short-term readmission in patients with AD cirrhosis and to guide clinicians to develop individualized treatments based on risk stratification.

## Introduction

Acute decompensated (AD) cirrhosis, defined as the acute development of ascites, hepatic encephalopathy, gastrointestinal hemorrhage, bacterial infections, or a combination of these factors, is the main cause of hospitalization and mortality in patients with cirrhosis (1–3). Owing to these complications, patients with decompensated cirrhosis are more frequently hospitalized and rapidly readmitted shortly after discharge. Hospital readmission is considered as a surrogate marker of the quality of healthcare delivery systems. Moreover, readmissions are associated with negative outcomes in patients and their families, and they have a significant impact on the overall costs of health care (4).

An estimated 27.1% of readmissions may be avoidable (5). To date, several studies have focused on assessing the predictors of readmission in decompensated cirrhosis (6–18). However, hospital readmission rates of patients with cirrhosis remain high, ranging from 10 to 50%, with a pooled estimate of 26% at 30 days and 21–71% at 90 days (19). The reason for these findings may be that the effective implementation of interventions requires not only understanding risk factors, but also identifying high-risk patients on the basis of highly accurate individualized risk predictive models, given that misleading risk estimates often lead to inappropriate treatment choices (20, 21). Therefore, the establishment of a model that can effectively predict and distinguish the individual risk of readmission remains an urgent medical requirement.

The current study was conducted to determine the readmission risk factors for patients with AD cirrhosis-related complications, to develop and temporally validate a nomogram to estimate the individual probability of readmission within 90 days, and to guide clinicians to develop individualized counseling programs and treatments based on risk stratification.

## Patients and Methods

### Population and Study Design

We conducted a multicenter retrospective prognostic study of inpatients with AD cirrhosis at six tertiary hospitals in Chongqing, China, including the Second Affiliated Hospital of Chongqing Medical University, Yong Chuan Hospital of Chongqing Medical University, Third Affiliated Hospital of Chongqing Medical University, University-Town Hospital of Chongqing Medical University, People's Hospital of Tong Liang District, and Southeast Hospital of Chongqing. We followed the transparent reporting of a multivariable predictive model for individual prognosis or diagnosis (TRIPOD) guidelines for model development and validation (22). Clinical data were collected using electronic medical record systems. Consecutive patients with AD cirrhosis admitted to the above hospitals from September 2012 to December 2016 were enrolled as the derivation cohort, and those with the same clinical characteristics hospitalized between January 2017 and April 2020 were enrolled as the temporal validation cohort. To determine whether patients admitted in April 2020 were readmitted to the hospital, we conducted follow-up until August 2020. The end points were cirrhosis-related readmissions within 90 days from the date of hospital discharge. In cases of multiple admissions, only the first readmission was considered.

The study protocol was reviewed and approved by the ethics committee of the First Affiliated Hospital of Chongqing Medical University. Due to its retrospective nature, this study required no conformed consent.

### Inclusion and Exclusion Criteria

The inclusion criteria were as follows: (1) patients aged ≥ 18 years and (2) hospital admission for AD cirrhosis.

The exclusion criteria were as follows: (1) patients with acute-on-chronic liver failure (ACLF): diagnosis of ACLF was based on the criteria from the consensus recommendation of the Asian Pacific Association for the Study of the Liver (23); (2) liver cancer or other active malignancies; (3) evidence of congestive heart failure, chronic kidney disease, or other significant chronic extrahepatic diseases; (4) hospital stay ≤ 1 day; (5) endoscopic ligation of esophageal varices or transjugular intrahepatic portosystemic shunt (TIPS) in the initial hospitalization or elective hospital admission; (6) discharge against medical advice; (7) patients lost to follow-up or death during index hospitalization; and (8) patients with >30% of data missing. Details of readmission in other hospitals or planned procedures, surgery, and therapy were compiled from medical history.

AD cirrhosis was defined as the rapid development of one or more major complications of liver disease, such as ascites, encephalopathy, gastrointestinal hemorrhage, bacterial infection, or a combination of these factors, requiring hospitalization (1, 24–27).

Ascites was recorded as the primary reason for admission if this was the sole criterion for admission, and infection was absent. Hepatic encephalopathy was characterized by altered mental status or neuropsychiatric abnormalities in the presence of liver cirrhosis after exclusion of other causes. Gastrointestinal (GI) bleeding was defined as the development of an upper and/or lower gastrointestinal hemorrhage of any etiology (27). Bacterial infection was defined in cases of spontaneous bacterial peritonitis, pneumonia, cellulitis, biliary tract infection, urinary system infection, and spontaneous bacteremia (26).

In the presence of more than one contributory factor, the main cause of admission was defined as follows: (1) in patients admitted with GI bleeding in the presence of ascites, bacterial infection, or hepatic encephalopathy, GI bleeding was considered the main cause; (2) in the absence of bleeding at admission, bacterial infection was the main cause of hospitalization; and (3) in patients with hepatic encephalopathy and ascites, the main cause was the former (11). The principal cause of hospitalization was subsequently assessed independently by two subspecialist physicians.

### Treatment

Medical therapies were used for all patients during hospitalization and after discharge, such as antiviral therapy, diuretics, lactulose, non-selective beta-blockers, antibiotics, symptomatic and supportive therapies. Prophylactic antibiotics were not routinely administered after discharge.

### Data Collection

Demographic, etiological, clinical, and laboratory data were recorded within 24 h of the first hospital visit. Demographic characteristics included age and sex. The etiological characteristics, including hepatitis B virus (HBV)/hepatitis C virus (HCV) infection, autoimmunity, and alcohol consumption, were assessed from medical history. Clinical data included length of hospital stay, complications related to liver cirrhosis, comorbidities, smoking history, alcohol consumption, and family history of liver disease. Laboratory analyses included liver function test, routine blood test, creatinine, blood urea nitrogen, serum sodium, serum potassium, and international normalized ratio (INR). End-stage Liver Disease score (MELDs), Child–Turcotte–Pugh score (CTPs), chronic liver failure-consortium acute decompensation scores (CLIF-C ADs), and MELD-Na score (MELD-Nas) were calculated at admission according to previously published criteria (28–31).

### Statistical Analyses

Continuous variables were expressed as mean ± standard deviation (SD) or median (interquartile range), according to the distribution of normality. Categorical variables were reported as counts with percentages. Group comparisons of continuous variables were analyzed with the Mann–Whitney *U* test and categorical variables with χ^2^ or Fisher's exact test. For variables with omission rates <30%, multiple imputation was used.

To avoid overfitting, we performed two steps of variable selection. First, we evaluated the association between readmission within 90 days and a set of potential predictors by using univariate analyses (Mann–Whitney *U* test for quantitative predictors and χ^2^ or Fisher's exact test for binary predictors). Predictors with *p* < 0.05 were subsequently considered in an automated variable selection procedure within the least absolute shrinkage and selection operator (LASSO) framework to select the best predictor subset (32). The complexity of LASSO regression was controlled by a tuning parameter lambda (λ) with the rule that the penalty for each variable coefficient increases with λ value, and the relevant features with non-zero coefficients were selected that contributed to the final LASSO regression (33). The number of variables involved in the final model was considered based on the optimal λ value to balance the accuracy and simplicity of the model. Then, the retained variables were used to construct the nomogram using multivariate Cox regression. The nomogram was based on the fitted predictive model using R version 4.0.2 with the rms package (34). To streamline the power calculation estimation, we produced PowerTools, an interactive open-source web application, written in R code by using the Shiny framework (http://www.shinyapps.io/).

The discriminatory value of the models was assessed based on the concordance index (C-index). The area under the curve (AUC) of the receiver operating characteristic (ROC) curve was also used to evaluate the prognostic accuracy of the nomogram. To demonstrate the stability of the model, we applied bootstraps with 200 resamples to correct the C-index to overcome overfitting. Calibration curves were additionally drawn to evaluate the concordance between the predicted and observed probabilities. The nomogram model was validated with a temporal validation cohort using the same process of capability assessment. Decision curve analyses (DCA) were applied to compare the benefits and improved performance of different models (35).

According to the nomogram score, the patients were classified into two groups representing low and high risk. The optimal cut-off values for the total points of the nomogram were determined by maximizing the Youden index (sensitivity + specificity – 1). Kaplan–Meier curves were constructed for stratified risk groups, and log-rank tests were used to determine significant differences between the curves.

All tests were two-sided, and data were considered statistically significant at *p* < 0.05. Statistical analyses were performed using R software (version 4.0.2, Vienna, Austria).

## Results

### Characteristics of the Study Cohort

A total of 8,402 patients met the inclusion criteria. Following the application of the exclusion criteria, 956 patients were finally included in the study, specifically 705 patients in the derivation cohort (from September 2012 to December 2016) and 251 patients in the temporal validation cohort (from January 2017 to April 2020). The study selection process is depicted as a flow chart (see Supplementary Figure 1 for details).

The mean (SD) age of all patients was 58.8 (12.6) years, and 68.31% were male. The etiologies of cirrhosis were chronic hepatitis B (50.4%), alcoholic (10.7%), autoimmune liver disease (9.9%), chronic hepatitis C (5.0%), and other/cryptogenic factors (19.6%). The overall readmission rates for patients at 30, 60, and 90 days were 24.6, 43.0, and 51.8%, respectively. As shown in Table 1, bacterial infection was the main reason for index admission (54.3%), followed by GI bleeding (35.0%), hepatic encephalopathy (5.8%), and ascites (4.9%). Regarding the main reason for readmission, bacterial infection was the most common cause, followed by GI bleeding, other cirrhosis-related diseases, hepatic encephalopathy, and ascites at the 30-, 60-, and 90-day time points.

**Table 1:** Cirrhosis-related index hospitalizations and readmissions.

	**GI bleeding**	**Bacterial infection**	**HE**	**Ascites**	**others**
Index, No. (%)	335 (35.0)	519 (54.3)	47 (4.9)	55 (5.8)	N/A
30-day, No. (%)	53 (22.6)	105 (44.7)	21 (8.9)	9 (3.8)	47 (20.0)
60-day, No. (%)	89 (21.7)	198 (48.2)	36 (8.8)	15 (3.7)	73 (17.8)
90-day, No. (%)	104 (21.0)	242 (48.9)	40 (8.1)	19 (3.8)	90 (18.2)

*GI, gastrointestinal; HE, hepatic encephalopathy; NA, not applicable*.

Based on the baseline characteristics of the two cohorts of patients as listed in Supplementary Table 1, patients in the derivation set were older and had lower neutrophil percentages and blood urea nitrogen levels; higher total protein, total bilirubin (TB), direct bilirubin, hemoglobin, aspartate aminotransferase, and alkaline phosphatase levels; lower rates of gastrointestinal hemorrhage; higher rates of bacterial infection; higher lengths of stay at initial admission; and higher CTPs and CLIF-C ADs (*p* < 0.05). The 30-, 60-, and 90-day risk of readmission were higher for the temporal validation set than for the derivation set (*p* < 0.05). The remaining clinical and laboratory parameters at initial admission as well as MELDs and MELD-Nas were similar between the derivation and temporal validation sets.

### Development of a Nomogram

Supplementary Table 2 provides the results of the univariate analyses for all 36 factors considered as potential predictors in our scoring system. Fifteen candidate predictor variables with *p* < 0.05 were used as the input data in the LASSO regression. When the lambda value was collected as 1 standard error [log (λ1se) = −2.10], four variables were selected (see Supplementary Figure 2 for details). Then, the four retained variables were used to construct the nomogram using multivariate Cox regression. As shown in Table 2, GI bleeding, serum sodium, total bilirubin (TB), and INR composed a panel of significant predictors of readmission in patients with AD cirrhosis.

**Table 2:** The HR values of the independent risk factors for prediction of 90-day readmission in patients with acute decompensated cirrhosis.

	**HR**	**95%CI**	***P*-value**
**GI bleeding**	2.787451	(2.2207,3.4988)	<0.001
**Serum sodium**	0.9552653	(0.9333,0.9777)	<0.001
**Total bilirubin**	1.0037945	(1.0026,1.0050)	<0.001
**INR**	1.3975204	(1.1264,1.7339)	0.002

*GI, gastrointestinal; INR, international normalized ratio; HR, hazard ratio; CI, confidence interval*.

**Figure 1:**
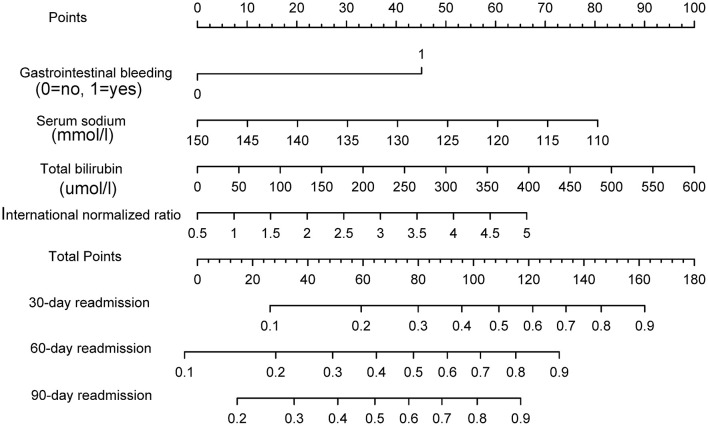
The nomogram to predict the risk of readmission in patients with acute decompensated cirrhosis.

A nomogram was constructed based on the four aforementioned independent prognosticators (Figure 1). The values of each risk factor were assigned a score on the point scale axis. By adding each single score and using that value in the total point scale axis, the total score could be easily calculated to assign the probability of readmission for individual patients at 30, 60, and 90 days. For the convenience of clinicians, we have provided the nomogram as a web-based calculator tool (https://cqykdx1111.shinyapps.io/dynnomapp/). Doctors can enter the indicators for each patient to automatically calculate the patient's probability of readmission within 90 days.

**Figure 2:**
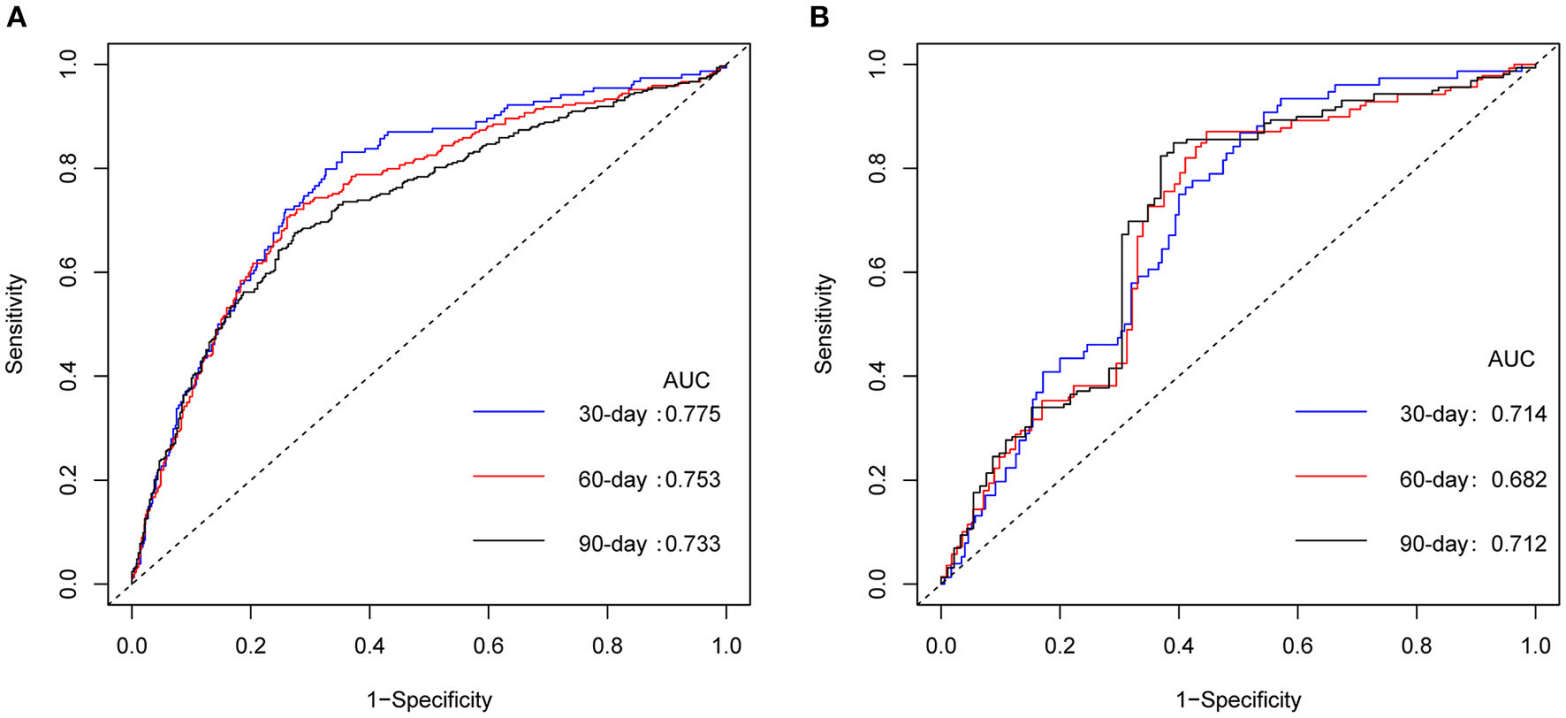
ROC curves for nomogram. ROC curves of nomogram in derivation cohort **(A)** and in temporal validation cohort **(B)**. ROC, receiver operating characteristic; AUC, area under the curve.

The model exhibited good discrimination ability. The C-index values of the nomogram for 30-, 60-, and 90-day readmission were 0.770 [95% confidence interval (CI): 0.728–0.812], 0.754 (95% CI: 0.716–0.791), and 0.731 (95% CI: 0.693–0.768), respectively. Furthermore, the predictive performance of nomogram was calculated by AUC of ROC curve. The AUC values were 0.775 (95% CI: 0.733–0.817), 0.753 (95% CI: 0.715–0.791), and 0.733 (95% CI: 0.695–0.770) for 30-, 60-, and 90-day, respectively (Figure 2A). Bootstraps with 200 resamples were performed to correct the predictive model. The adjusted C-index values of the nomogram for 30-, 60-, and 90-day readmission were 0.770, 0.750, and 0.732, respectively.

**Figure 3:**
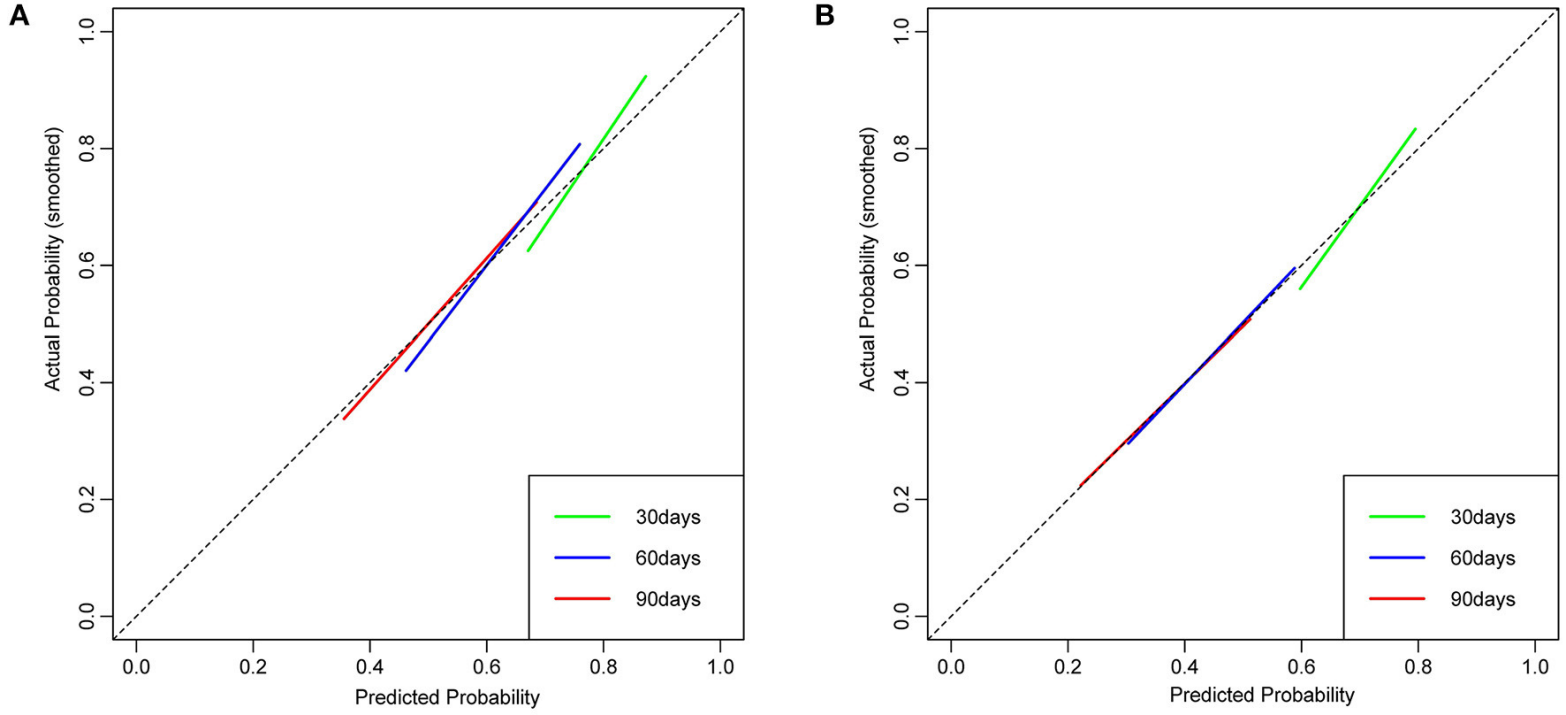
The calibration curve of nomogram at 30, 60, and 90 days for the derivation cohort **(A)** and the temporal validation cohort **(B)**. Dashed lines along the 45-degree line through the point of origin represent the perfect calibration models in which the predicted probabilities are identical to the actual probabilities.

The calibration curves showed good agreement between nomogram predictions and observed probabilities for 30-, 60-, and 90-day outcomes in the derivation cohort (Figure 3A).

### Temporal Validation of the Nomogram

The validation set was estimated using the established nomogram, and the C-index values obtained were 0.703 (95% CI 0.638–0.768), 0.694 (95% CI: 0.627–0.762), and 0.707 (95% CI: 0.636–0.777), respectively. The AUC values were 0.714 (95% CI: 0.649–0.778), 0.682 (95% CI: 0.614–0.751), and 0.712 (95% CI: 0.644–0.780), respectively (Figure 2B), supporting the suitability of the nomogram for estimating 30-, 60-, and 90-day readmission.

The calibration curves revealed good agreement between the nomogram predictions and observed probabilities for 30-, 60-, and 90-day outcomes in the temporal validation cohort (Figure 3B).

### Comparison of Predictive Accuracy for Readmission Among Nomogram, MELDs, CLIF-C ADs, CTPs, and MELD-Nas

Predictive power for readmission was compared for the nomogram, MELDs, CLIF-C ADs, CTPs, and MELD-Nas based on C-indexes. DCA was performed to determine the clinical utility of the nomogram by calculating the net benefits at different threshold probabilities. The C-indexes for 30-, 60-, and 90-day readmission predicted with MELDs, CLIF-C ADs, CTPs, and MELD-Nas were significantly lower than those with the nomogram, in both the derivation and temporal validation cohorts (Table 3).

**Table 3:** Predictive discrimination ability of nomogram as compared to MELDs, CLIF-C ADs, CTPs, and MELD-Nas in the derivation and temporal validation cohort.

	**Nomogram** **C-index** **95%CI**	**MELDs** **C-index** **95%CI**	**CLIF-C ADs** **C-index** **95%CI**	**CTPs** **C-index** **95%CI**	**MELD-Nas** **C-index** **95%CI**
**Derivation cohort** ***N*** **= 705**					
30-day	0.77 (0.728,0.812)	0.659 (0.610,0.709)	0.678 (0.630,0.727)	0.638 (0.587,0.690)	0.674 (0.624,0.723)
*P*-value vs. readmission		<0.001	<0.001	<0.001	<0.001
60-day	0.754 (0.716,0.791)	0.621 (0.578,0.663)	0.655 (0.614,0.697)	0.598 (0.555,0.641)	0.649 (0.608,0.691)
*P*-value vs. readmission		<0.001	<0.001	<0.001	<0.001
90-day	0.731 (0.693,0.768)	0.609 (0.567,0.650)	0.662 (0.622,0.702)	0.604 (0.563,0.645)	0.647 (0.606,0.687)
*P*-value vs. readmission		<0.001	<0.001	<0.001	<0.001
**Validation cohort** ***N*** **= 251**					
30-day	0.703 (0.638,0.768)	0.574 (0.495,0.652)	0.619 (0.543,0.696)	0.590 (0.514,0.666)	0.606 (0.528,0.683)
*P*-value vs. readmission		<0.001	<0.001	0.029	<0.001
60-day	0.694 (0.627,0.762)	0.569 (0.498,0.640)	0.558 (0.487,0.629)	0.627 (0.559,0.696)	0.520 (0.449,0.592)
*P*-value vs. readmission		<0.001	<0.001	0.029	<0.001
90-day	0.707 (0.636,0.777)	0.607 (0.537,0.677)	0.599 (0.528,0.669)	0.649 (0.579,0.718)	0.556 (0.484,0.628)
*P*-value vs. readmission		<0.001	<0.001	0.029	<0.001

*95% CI, 95% confidence interval; MELDs, model for end-stage liver disease score; CLIF-C ADs, chronic liver failure-consortium acute decompensation scores; CTPs, child-turcotte-pugh score; MELD-Nas, MELD-Na score*.

**Figure 4:**
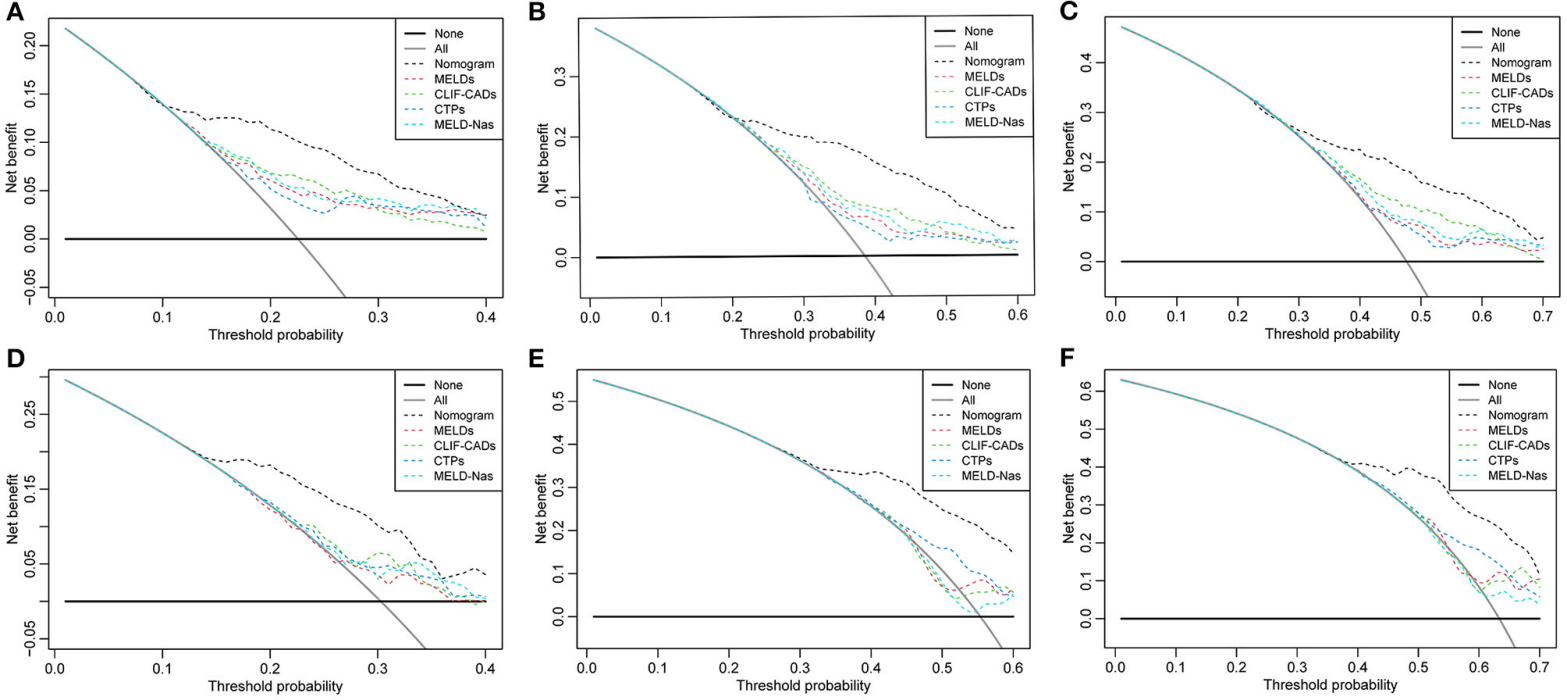
Decision curve analysis at 30, 60, and 90 days for the derivation cohort **(A–C)** and the temporal validation cohort **(D–F)**. The horizontal solid black line represents the assumption that no patients will experience the event, and the solid gray line represents the assumption that all patients will relapse. On decision curve analysis, the readmission nomogram showed superior net benefit compared with other models across a range of threshold probabilities.

Using DCA, our nomogram provided superior net benefit and displayed improved performance in prognostic evaluation over the 30-, 60-, and 90-day periods, both in the derivation (Figures 4A–C) and validation (Figures 4D–F) cohorts, compared to MELDs, CLIF-C ADs, CTPs, and MELD-Nas models.

### Performance of the Nomogram in Stratifying Patient Risk

**Figure 5:**
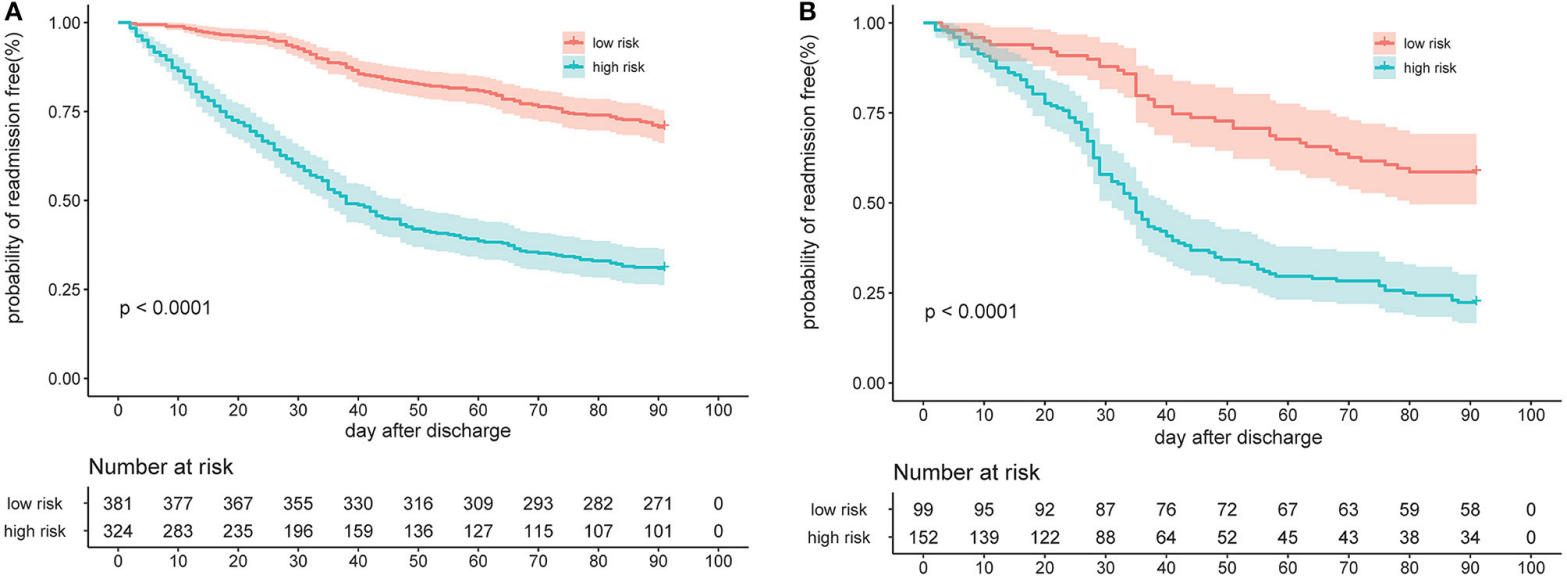
Risk group stratification according to bisection of the nomogram predicted readmission in the derivation cohort **(A)** and the temporal validation cohort **(B)**.

When patients were stratified according to the optimal cut-off value by the total nomogram points (high risk: >56.8 and low risk: ≤ 56.8), each group represented a distinct prognosis. The high-risk group was more likely to have readmission than the low-risk group, with statistical significance in both the derivation cohort and temporal validation cohort (*p* < 0.0001, Figures 5A,B).

## Discussion

In the present study, we generated an easy-to-perform nomogram consisting of clinical complications and laboratory indicators for the first time that could be effectively used to prognosticate the readmission probability of AD cirrhotic patients receiving drug-based therapy at different time points within a 90-day period. To avoid the effects of other coexisting medical conditions, we only included patients with cirrhosis-related complications as the reason for initial hospitalization or readmission. The nomogram model performed well, as determined from C-indexes, AUC values, and the calibration curves both in the derivation cohort and temporal validation cohort at 30, 60, and 90 days, respectively. According to DCA, our nomogram demonstrated better net benefit and improved performance in 30-, 60-, and 90-day prognostic evaluations in both the derivation and validation cohorts, compared with MELDs, CLIF-C ADs, CTPs, and MELD-Nas. Furthermore, the model was able to stratify patients into groups with high and low risk of readmission within 90 days. Finally, our nomogram is accessible to medical staff via a link to the algorithm to automatically calculate a patient's probability of readmission within 90 days (https://cqykdx1111.shinyapps.io/dynnomapp/). This scoring system can facilitate early identification of high-risk patients, thus allowing implementation of interventions during hospitalization to reduce readmission.

Readmission among patients with AD cirrhosis in the current study was common, with incidence rates of 24.6, 43.0, and 51.8% at 30, 60, and 90 days, respectively. This finding was similar to that obtained from earlier studies based in India and North America (10, 16, 18). The etiology of cirrhosis in India was mainly hepatitis B virus (50.4%), while in North America and Europe, the main causes of cirrhosis were alcohol consumption (29.4%) and HCV (39.3%) (10, 11). We identified bacterial infection as the main reason for index admission and readmission (54.3% at initial admission, 44.7% at 30 days, 48.2% at 60 days, and 48.9% at 90 days), distinct from findings from India, North America, and Europe, where hepatic encephalopathy and ascites were identified as the main contributory factors (10, 11, 16, 18, 36). This difference may be associated with the distinct inclusion/exclusion criteria and medical conditions in different regions. The admission of cirrhosis patients with bacteremia to the intensive care unit was associated with an increase in the severity of the disease and an increase in the need for extrahepatic organ support. Bacteremia was an independent predictor of mortality in patients with ACLF (37, 38). Recent novel perspectives in the management of decompensated cirrhosis suggest that the systemic inflammatory response is one of the upstream events underlying the development of complications of liver cirrhosis (39). A PREDICT study showed that the most severe course of acute decompensation occurs in patients with pre-ACLF who display rapid progression of systemic inflammation leading to the development of ACLF and death within 90 days (40). These findings clearly suggest that systemic inflammatory response is predictive of poor prognosis. Therefore, clinicians should pay significant attention to the prevention of infections, which could avoid downstream complications (further decompensation, repeat infections, ACLF, or death) of cirrhosis (41).

Our nomogram includes laboratory and clinical indicators, which is better compared with other models, reflecting the severity of disease. Hyponatremia has been associated with hepatorenal syndrome and ascites and is an important predictor of readmission and mortality among patients with decompensated cirrhosis (30, 42). INR and TB are critical markers of liver protein synthesis function and the extent of hepatocellular necrosis (23, 43). These three indicators are all or partly involved in the construction of CLIF-C ADs, MELD-Nas, MELDs, and CTPs, reported to be significantly associated with the prognosis of AD cirrhosis or readmission (6, 11, 16, 18, 31, 36). However, owing to the involvement of the logarithm in calculations of MELDs, MELD-Nas, and CLIF-C ADs, clinicians have to use calculators, making it impractical in busy clinical practice. Although the calculation of CTPs is relatively simple, there are still some limitations, such as the narrow range of disease severity and subjective criteria, including hepatic encephalopathy and ascites (44). Notably, our model was more accurate than CLIF-C ADs, MELD-Nas, MELDs, and CTPs in predicting the risk of AD cirrhosis readmission. GI bleeding is a frequent and serious complication of cirrhosis. Mortality rates associated with acute esophageal variceal bleeding (EVB) are 12–20% and as high as 50% with EVB rebleeding (45). We found that although GI bleeding was a risk factor, the main cause for readmission in our study was bacterial infection. This finding underscores that patients with decompensated cirrhosis with gastrointestinal bleeding may be more likely to develop community-acquired infections after being discharged. Further studies are needed to confirm this possibility.

Our study showed that the readmission rate of AD cirrhosis patients in the low-risk group with total points ≤ 56.8 was significantly lower than that of high-risk patients with total points >56.8. In situations of limited medical resources and to improve cost effectiveness, low-risk patients could be considered for early discharge, whereas high-risk patients, especially those with GI bleeding, might need intensive management to prevent short-term readmission.

Our study has several strengths. First, we included only patients readmitted to the hospital for the first time after the initial discharge, thus avoiding the effects of multiple admissions. Second, our study was based on the Cox proportional hazard model, which predicts the probability of readmission at different time points within 90 days, rather than a logistic model that predicts readmission risk at a single time point, as used in most previous studies. In addition, we used temporal validation to demonstrate that our model can be generalized to further applications.

## Limitations

This study has several limitations. First, selection bias may exist due to the retrospective nature of the investigation. However, we used a relatively large training cohort to construct the model, which was further subjected to temporal validation. Second, we excluded planned readmissions. Most of these patients underwent endoscopic variceal ligation during the initial hospitalization. Therefore, we did not evaluate the effects of this intervention on readmission. However, because the main reason for readmission is bacterial infection, the treatment target for patients with GI bleeding should not be limited to only preventing rebleeding. Third, data on social support, level of education, and socioeconomic status were not available. Further research is warranted to explore the impact of these important indicators.

## Conclusions

The rates of short-term readmission related to cirrhosis were high in our patients. Bacterial infection was the main cause of index admission and readmission. We developed and temporally validated a prognostic model that accurately predicts the incidence of cirrhosis-related readmissions in patients with AD cirrhosis receiving drug-based therapy. The readmission probability can be obtained with the nomogram scoring system, which is based on four independent variables for each patient (https://cqykdx1111.shinyapps.io/dynnomapp/). The present nomogram can assist in clinical decision-making, counseling for treatment, and, most importantly, risk stratification of patients to help differentiate patients who need intensive management to prevent short-term readmission from those who could discharge earlier.

## Data Availability Statement

The original contributions presented in the study are included in the article/Supplementary Material, further inquiries can be directed to the corresponding author/s.

## Ethics Statement

The studies involving human participants were reviewed and approved by The Ethics Committee of the First Affiliated Hospital of Chongqing Medical University. Written informed consent for participation was not required for this study in accordance with the national legislation and the institutional requirements.

## Author Contributions

XX, JT, and BQ: concept and design. XX and JT: drafting of the manuscript. XX, JT, HW, and WZ: statistical analysis. HW, WZ, and BQ: administrative, technical, and material support. XX and BQ: had full access to all of the data in the study and take responsibility for the integrity of the data and the accuracy of the data analysis and supervision. All authors critical revision of the manuscript for important intellectual content, acquisition, analysis, or interpretation of data, and read and approved the final manuscript.

## Conflict of Interest

The authors declare that the research was conducted in the absence of any commercial or financial relationships that could be construed as a potential conflict of interest.

## Abbreviations

AD: acute decompensated
TRIPOD: transparent reporting of a multivariable prediction model for individual prognosis or diagnosis
ACLF: acute-on-chronic liver failure
TIPS: transjugular intrahepatic portosystemic shunt
GI: gastrointestinal
HBV: hepatitis B virus
HCV: hepatitis C virus
INR: international normalized ratio
MELDs: model for end-stage liver disease score
CTPs: Child-Turcotte-Pugh score
CLIF-ADs: chronic liver failure-consortium acute decompensation scores
MELD-Nas: MELD-Na score
SD: standard deviation
LASSO: least absolute shrinkage and selection operator
λ: lambda
AUC: area under the curve
ROC: receiver operating characteristic
C-index: concordance index
DCA: decision curve analyses
TB: total bilirubin
CI: confidence interval
EVB: esophageal variceal bleeding.
