# Supplementary material for: Risk Stratification Score to Predict Readmission of Patients With Acute Decompensated Cirrhosis Within 90 Days
Source: Front Med (Lausanne). 2021 May 31;8:646875. doi: 10.3389/fmed.2021.646875 (PMC8200567; doi:10.3389/fmed.2021.646875)

Supplementary information

Figure S1 Flow of inclusions and exclusions

Table S1 Baseline characteristics of patients in the derivation and temporal validation cohort

Table S2 Univariable analysis of factors in patients readmission and non-readmission within 90 days in derivation cohort

Figure S2 A graph showing the cross validation in LASSO regression to screen variables

Figure S1 Flow of inclusions and exclusions

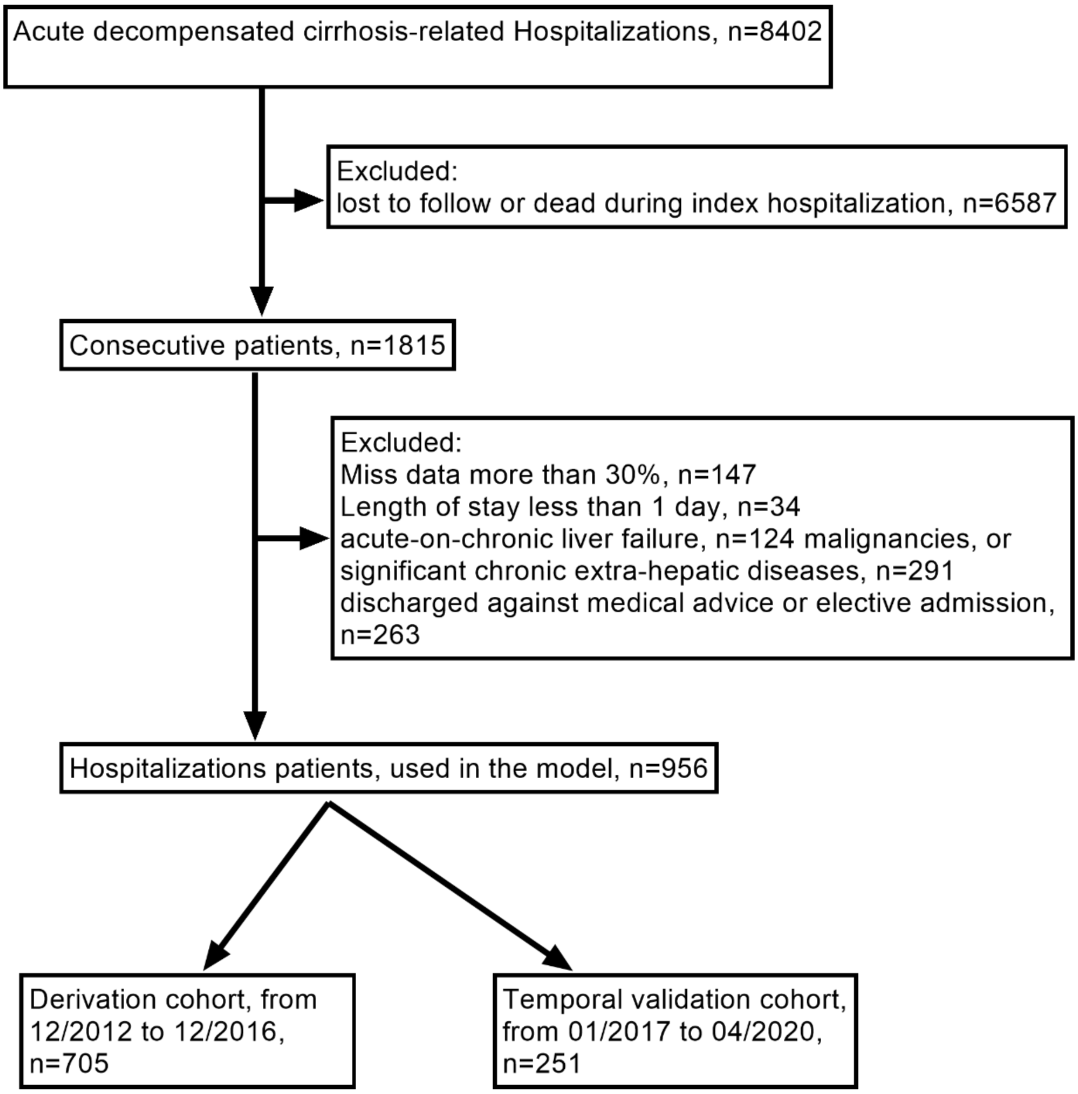

Table S1 Baseline characteristics of patients in the derivation and temporal validation cohort

Variables	Derivation N=705	Temporal validation N=251	P value
Age (years), mean (SD)	59.6 (12.5)	56.7 (12.6)	0.006
Gender, male, No. (%)	469(66.5)	184(73.3)	0.057
**Etiology, No. (%)**			<0.001
HBV	358(50.8)	124(49.4)	0.763
HCV	35(5.0)	13(5.2)	1.000
Alcoholism	82(11.6)	20(8.0)	0.135
Autoimmune hepatitis	80(11.4)	15(6.0)	0.020
HBV +alcoholism	37(5.3)	5(2.0)	0.047
other/cryptogenic	113 (16.0)	74 (29.5)	<0.001
**Clinical status, No. (%)**			
GI bleeding	200(28.4)	135(53.8)	<0.001
Bacterial infection	428(60.7)	91(36.3)	<0.001
Ascites	32(4.5)	15(6.0)	0.463
Hepatic encephalopathy	45(6.4)	10(4.0)	0.214
Hypertension	74(10.5)	37(14.7)	0.091
Cardio-cerebrovascular disease	37(5.3)	21(8.4)	0.105
Diabetes	144(20.4)	40(15.9)	0.145
Smoking history	312(44.3)	116(46.2)	0.644
Alcohol consumption	318(45.1)	118(47.0)	0.655
Family history of liver disease	141(20.0)	48(19.1)	0.836
Length of index hospitalization median (IQR)	14 (9, 20)	11 (7, 15)	<0.001
30-day readmission, No. (%)	159(22.6)	76(30.3)	0.018
60-day readmission, No. (%)	272(38.6)	139(55.4)	<0.001
90-day readmission, No. (%)	336(47.7)	159(63.4)	<0.001

**Laboratory parameters, median(IQR)**			
Total protein (g/L)	64 (58.3, 70.1)	61.5 (55.5, 67.5)	<0.001
Albumin (g/L)	30.4 (27, 34.1)	30.7 (27.4, 34.4)	0.587
Total bilirubin (μmol/L)	29.1 (17, 52.9)	23 (14.8, 48.1)	0.015
Direct bilirubin (μmol/L)	14.6 (8.5, 30.6)	10.4 (6.2, 22.3)	<0.001
Alanine aminotransferase (U/L)	32 (21, 56)	31 (20.3, 54.3)	0.530
Aspartate aminotransferase (U/L)	50 (33, 87)	44.3 (30, 74.5)	0.032
Alkaline phosphatase (U/L)	109 (76, 156)	97.9 (70, 134.5)	0.005
γ–glutamyl transferase (U/L)	52 (27, 122)	50 (28, 110.5)	0.706
White blood count (10^9/L)	4.3 (3, 6.4)	4.3 (2.9, 6.5)	0.763
Neutrophil percentage (%)	69.4 (61.1, 77.2)	72 (61.6, 78.8)	0.020
Blood platelet (10^9/L)	65 (45, 100)	64 (43, 95.5)	0.333
Hemoglobin (g/L)	101 (79, 120)	93 (75.5, 112)	0.002
Serum sodium (mmol/L)	139.2 (136.3,141.5)	138.5 (136, 141.5)	0.176
Serum potassium (mmol/L)	3.8 (3.4, 4.1)	3.8 (3.4, 4.2)	0.844
International normalized ratio	1.4 (1.2, 1.6)	1.4 (1.2, 1.6)	0.871
blood urea nitrogen (mmol/L)	5.2 (4.1, 7.1)	6 (4.4, 8.3)	<0.001
Serum creatinine (μmol/L)	66.9 (56.3, 79)	65.6 (55, 76.8)	0.162
MELDs	11 (8.1, 15.2)	11.2 (9.2, 14.2)	0.330
CLIF-C ADs	23.2 (20.9, 26.2)	22.4 (20.2, 25.5)	0.006
CTPs	7 (6, 9)	7 (6, 8)	0.013
MELD-Nas	12.4 (8.7, 17.8)	12.8 (10, 17.3)	0.182

Abbreviations: SD, standard deviation; HBV, hepatitis b virus; HCV, hepatitis c virus; GI, gastrointestinal; IQR, interquartile range; MELDs, model for End-stage Liver Disease score; CLIF-C ADs, chronic liver failure-consortium acute decompensation scores; CTPs, Child-Turcotte-Pugh score; MELD-Nas, MELD-Na score

Table S2 Univariable analysis of factors in patients readmission and non-readmission within 90 days in derivation cohort

Variables	Readmission n=336	Non-readmission n=369	P value
Age (years), mean (SD)	58.36±11.91	60.68±12.99	0.011
Gender, male, No. (%)	235(69.9)	234(63.4)	0.067
**Etiology, No. (%)**			
HBV	183(54.5)	175(47.4)	0.062
HCV	8(2.4)	27(7.3)	0.003
Alcoholism	39(11.6)	43(11.7)	0.985
Autoimmune hepatitis	29(8.6)	51(13.8)	0.030
HBV + alcoholism	17(5.1)	20(5.4)	0.83
other/cryptogenic	60(17.9)	53(14.4)	0.207
**Clinical status, No. (%)**			
GI bleeding	133(39.6)	67(18.2)	<0.001
Bacterial infection	167(49.7)	261(70.7)	<0.001
Ascites	9(2.7)	23(6.2)	0.024
Hepatic encephalopathy	27(8.0)	18(4.9)	0.087
Hypertension	34(10.1)	40(10.8)	0.755
Cardio-cerebrovascular disease	16(4.8)	21(5.7)	0.581
Diabetes	71(21.1)	73(19.8)	0.658
Smoking history	157(46.7)	155(42.0)	0.208
Alcohol consumption	154(45.8)	164(44.4)	0.711
Family history of liver disease	65(19.4)	76(20.6)	0.678
Length of index hospitalization median (IQR)	14(9,22)	14(9,19)	0.126
**Laboratory parameters, median (IQR)**			
Total protein (g/L)	62.8(57.2, 69.1)	65.3(60.2, 71.9)	0.001
Albumin (g/L)	29.8(26.5, 33.4)	31(27.4, 34.8)	0.019
Total bilirubin (μmol/L)	30.3(17.3, 77.9)	28.3(16.9, 44)	0.018
Direct bilirubin (μmol/L)	15.5(8.8, 47.8)	13.9(8.2, 24.7)	0.014

Alanine aminotransferase (U/L)	34(22, 58.3)	31(20, 53)	0.109
Aspartate aminotransferase (U/L)	51(34, 90.3)	48(32, 82)	0.179
Alkaline phosphatase (U/L)	109(75, 155.3)	108(77, 156)	0.86
γ–glutamyltransferase (U/L)	47.6(26, 109.6)	54(28, 129)	0.159
White blood count (10^9/L)	4.4(3.0, 6.8)	4.2(3.0, 6.0)	0.21
Neutrophil percentage (%)	70.2(62.1, 77.6)	68.6(59.9, 76.9)	0.138
Blood platelet (10^9/L)	67(45, 98.3)	64(45, 100)	0.764
Hemoglobin (g/L)	98(77, 117.3)	104(82, 122)	0.041
Serum sodium (mmol/L)	138.4(135.2, 140.8)	139.9(137.2, 142)	<0.001
Serum potassium (mmol/L)	3.8(3.4, 4.1)	3.8(3.4, 4.1)	0.319
International normalized ratio	1.4(1.2, 1.8)	1.3(1.2, 1.6)	<0.001
blood urea nitrogen (mmol/L)	5.3(4.1, 7.5)	5.1(4.0, 6.8)	0.027
Serum creatinine (μmol/L)	69.0(57.9, 81.1)	64.7(54.7, 76)	0.003

Abbreviations: SD, standard deviation; HBV, hepatitis b virus; HCV, hepatitis c virus; GI, gastrointestinal; IQR, interquartile range;

Figure S2 A graph showing the cross validation in LASSO regression to screen variables

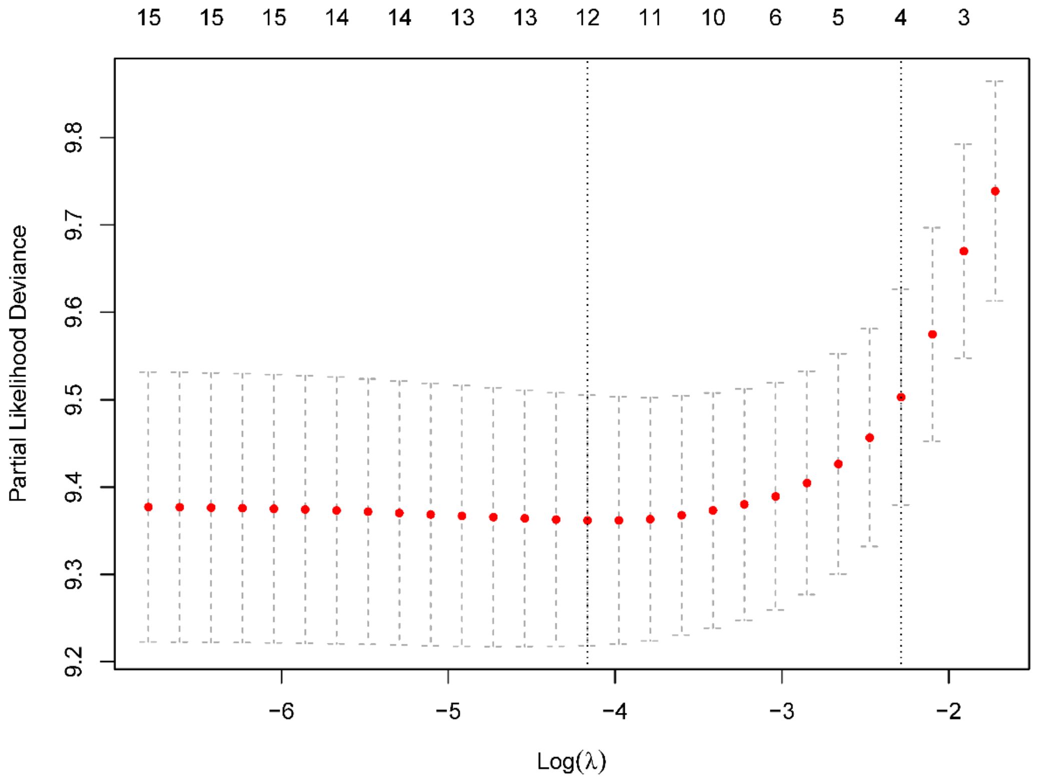

Supplement: Supplementary file 1 [file Data_Sheet_1.pdf]
